# Imaging approach to deposition and neurogenic arthropathies

**DOI:** 10.1007/s11604-024-01677-2

**Published:** 2024-11-02

**Authors:** Ricardo Cuervo Arevalo, Felipe Aluja-Jaramillo, Carlos Corredor, Juan José Ramirez

**Affiliations:** Pontificia Universidad Javeriana, Hospital Universitario San Ignacio, Carrera 7 No 40 – 62, 110231 Bogotá, Colombia

**Keywords:** Arthropathy, Neurogenic, Radiography, Crystal arthropathies, Gout, Musculoskeletal diseases

## Abstract

Deposition arthropathies are a heterogeneous group of diseases in which organic and inorganic substances accumulate in different regions of the body with significant complications and morbidity. Although they are clearly distinct entities, the clinical manifestations are similar, so that joint evaluation by imaging provides a more accurate diagnostic approach by identifying findings characteristic of certain pathologies and is considered the gold standard in certain conditions. However, neuropathic arthropathy, especially Charcot’s foot, is an essential differential diagnosis in the joint evaluation of these patients because, although clinically similar, it manifests differently on radiological evaluation, with different treatment and prognosis. It is essential to know the main findings of the different imaging modalities, as well as their surgical performance, to propose the ideal study and differentiate these conditions with certainty.

## Introduction

Depositional diseases are a group of entities in which a variety of biochemical substances accumulate in different regions of the body, including the accumulation of precipitated crystals and other organic substances such as homogentisic acid and amyloid, or inorganic substances such as iron or copper [1]. These entities affect different age groups, most commonly adults over 50 years of age, with variable clinical manifestations and difficult diagnosis [2–4]. Lack of treatment leads to a wide spectrum of mainly neurological and joint complications [5, 6].

Neuropathic arthropathy is a predominantly bone-productive disease with a variety of causes, mainly neuropathic, traumatic and metabolic [1, 3, 7]. It usually manifests in people with diabetes mellitus and/or peripheral neurological disease through inflammatory changes and joint deformities, so its diagnosis is easily confused with biomechanical, infectious consequences or aggressive treatments such as amputation [3, 7–9]. The aim of this manuscript is to provide an overview of the most common deposition diseases; monosodium urate crystal deposition disease or gout (GO), calcium pyrophosphate (CPPD) chondrocalcinosis (CDC) and hydroxyapatite (HP), as well as neuropathic arthropathy of the foot or Charcot (CF), focusing on the imaging findings of the four modalities, ultrasonography (US), radiography (Rx), computed tomography (CT) and magnetic resonance (MR), especially high-resolution susceptibility images (HD-SWI), and providing the main operative parameters (Dx) involved in these diseases, such as sensibility (S) and specificity (SP).

### Anatomy of the synovial joint and normal imaging appearance

The synovial joint consists of a fibrous capsule lined internally by synovial tissue, which encloses two bony epiphyses, hyaline cartilage and a small cartilaginous area between the insertion of the capsule and the hyaline cartilage, known as the bare area. This area is lined with synovial tissue, making it susceptible to bony destruction by synovitis. The synovial joint is held in shape by passive stabilisers, ligaments (extensions of the joint capsule with insertions into the enteisis), and active stabilisers, tendons and muscles [1]. The synovial tissue also lines the tendon sheaths and bursae and produces fluid that nourishes and lubricates the hyaline cartilage and tendons [1]. It should be noted that normal synovial membrane is not visualised by imaging unless it is thickened, but ligaments can be identified directly by ultrasound and MR and indirectly by arthrography [1, 10].

### Indications for the main diagnostic imaging modalities

The use of multiple imaging modalities is beneficial for the depiction of deposition diseases and the characterization of their respective characteristics. It is important to recognise the advantages and disadvantages of each method to provide useful information for the clinician [10–12].Sensitivity and specificity of each method is described in Table 1.

**Table 1:** Diagnostic performance of imaging in deposition arthropathies and Charcot foot

	Plain Radiography	Ultrasonography	Tomography—DECT	Magnetic Resonance
Gout	CG: S 31%–SP 93% [15]	DC: S 83%–SP 76% [21] CG: S 48%–SP 96% [22]	Dx: S 78–100%–SP 89–100% [24]	Soft tissue [14]
CPPD	CDC: S 60%–SP 96% [16, 17]	Ps-DC: S 97.6%–SP 50.8% [39] Dx: S 81–88%–SP 59–92% [17]	Dx: S 78–100%–SP 94% [17]	Soft tissue [14]
Hydroxyapatite	High sensitivity [25]	Rotator cuff: S 98%–SP 94% [25]	More sensitive than the Rx [25]	S 65%–SP 58% [26] HD-SWI: S 98%–SP 96% [27]
Neuropathic arthropathy	S and SP < 50% [18, 19]	Complement study [18]	CNA: Preoperative. [18]	S 90%–SP 79% [18]

*S* sensitivity, *SP* specificity, *CG* chronic gout, *DC* double contour, *Ps-DC* pseudo double contour, *DX* diagnosis, *CDC* chondrocalcinosis, *ANA* acute neuropathic arthropathy, *OM* osteomyelitis, *CNA* chronic neuropathic arthropathy, *HD-SWI* high definition susceptibility images, *Rx* radiography

#### Plain films

In cases of chronic gout (CG), plain radiography may be useful in searching for evidence of crystal deposition [11–14]. In the event that HP is suspected, plain films are the initial modality. In cystic fibrosis, plain radiography is a useful initial modality and follow-up procedure. The test has a low sensitivity and specificity for the detection of early findings. The ability of plain films to differentiate between osteomyelitis and other conditions is less than 50% [18, 19].

#### Ultrasonography

Ultrasound is considered the initial investigative method in GO [20]. It is useful in determining the location of the crystal, with the double outline sign demonstrating a sensitivity of 83% and a specificity of 76% [21]. Gouty tophus may be observed in superficial soft tissue, with a sensitivity of 48% and a specificity of 96% [22]. In contrast, ultrasound has a higher sensitivity than radiographs, ranging from 81 to 88% [17, 23]. In cases of HP, ultrasound is useful for echo-guided treatment and also has a higher sensitivity of 98% and a higher positive predictive value of 94% for rotator cuff involvement [25]. In the context of CF, it is regarded as a supplementary approach for the assessment and delineation of soft tissue involvement [18, 19].

#### Tomography—DECT

Dual energy computed tomography (DECT) is the GOLD standard for diagnosis and follow-up of patients with S 78–100% and SP 89–100% [24]. In CPPD, it can differentiate basic calcium deposits from monosodium urate deposits by their density, with S 78–100% and SP 94% [18]. Basic calcium deposits tend to be more hyperdense, 450 Hounsfield units (HU), than monosodium urate deposits [16]. In HP deposits, tomography is clearly superior to conventional radiography in identifying erosions and calcium migration [25]. It also distinguishes between basic calcium deposits (100–400 HU) and ossifications (700–1500 HU) by their density [25]. Finally, in the case of CF, it describes bone erosions and sclerosis [18].

#### Mangetic resonance

MR detects joint effusion, synovitis, bone oedema, soft tissue changes and crystal deposition in virtually all diseases [14]. In the setting of CPPD, its diagnostic value is limited [14, 16], but high definitión susceptibility images sequences (HD-SWI) are attempted to identify and quantify monosodium calcium deposits [14]. In HP deposits, crystal displacement and resultant inflammation can be identified [25]. However, when used in isolation, basal calcium deposits can be confused with other structures or even missed, giving them an S 65% and SP 58% [26]. However, HD-SWI sequences can diagnose and exclude calcific tendinitis in patients with chronic shoulder pain, with a S of 98% and an SP of 96% [26, 27]. In the case of CF, its main use is to exclude differential diagnoses, mainly osteomyelitis, with an S 90 and E 79% [18].

### Semiological approach and the ABCDE’S of arthritis

The imaging approach should be global and regional [1]. The location is considered a key data point in differentiating between arthropathies, classifying them according to their number, mono, oligo, polyarticular, type of joint, small or large, and whether they depend on the axial or appendicular skeleton [1]. It should also identify which joint components and pararticular structures are involved, including cartilage, synovium, fluid, tendons, muscles and ligaments [1, 28]. The joint relationship, state of bone mineralisation, erosive and productive processes, compromise and manifestations in the surrounding soft tissues and associated findings should be reviewed in detail [1, 28].

To help remember the structures and findings to be considered in the imaging assessment, there is an acronym ‘ABCDE’S’ for plain radiography in the evaluation of arthropathies, which aims to categorise the joint processes as erosive, productive as in Charcot foot, or mixed as in crystal deposition disease [1]. The acronym and the most common findings in the main deposition diseases and Charcot foot are listed below:

- **A**lignment: Malalignment is often seen due to ligament laxity.
- **B**one: Increased local bone density due to reactive and reparative processes; osteophytes and enthesophytes.
- **C**artilage: Uniform loss of joint space in CPPD deposition. However, in GO the joint space is preserved without loss of cartilage.
- **D**istribution: Usually mono- and oligoarticular (up to 3 joints), symmetrical in CPPD and asymmetrical in GO.
- **E**rosions: Focal loss of subcortical bone seen in at least two planes [29, 30]. They are classified as marginal, juxta-articular or NON-marginal and subchondral or central;
- **S**oft tissue: Periarticular tophi and calcifications.

Common findings in the major inflammatory deposition arthropathies, especially on US, are joint effusion, synovitis, tenosynovitis and enthesitis.

## Major atrophies due to crystal deposition

### Gout or deposition of monosodium urate crystals

#### Definition, epidemiology and pathophysiology

The most common depositional arthropathy resulting from the deposition of monosodium urate crystals in and around the joints. It has a prevalence of between 0.1% and 10% in the world population and is more common in older men [2].

Primary or secondary hyperuricaemia predisposes to the formation of monosodium urate crystals around the joints, in the synovial fluid, enthesis or bone, leading to a local inflammatory response, synovitis and the formation of gouty tophi [14]. Gouty tophi are accumulations of crystals, proteins and lipids, surrounded by a granulomatous foreign body reaction, located in the hypodermis, joint or parapatellar space and tendons, most commonly the Achilles tendon, extensor mechanism and popliteus tendon [13, 20, 28]. However, there is a predilection for the first metatarsophalangeal joint of the foot with approximately 90% monoarticular presentation [14, 24].

#### Clinical presentation and general diagnostic approach

In clinical practice, it presents in four phases: asymptomatic, acute, intercritical and chronic. Acute crises are manifested by local inflammation and increased joint volume associated with systemic symptoms [21]. In the chronic phase, joints are bulky with nodules that may ulcerate and excrete whitish particulate material [24].

The GOLD standard for diagnosis is polarising microscopy, which identifies monosodium urate crystals after puncture of joint fluid or gouty tophi [21, 24]. However, the American College of Rheumatology and the European League Against Rheumatism have proposed diagnostic probability criteria using clinical, laboratory and imaging findings. A score > 8 provides specificity when imaging evaluation includes dual-energy US and CT scan for joint/bursa involvement or the presence of erosions on plain radiography [29].

#### Imaging approach

**Fig. 1:**
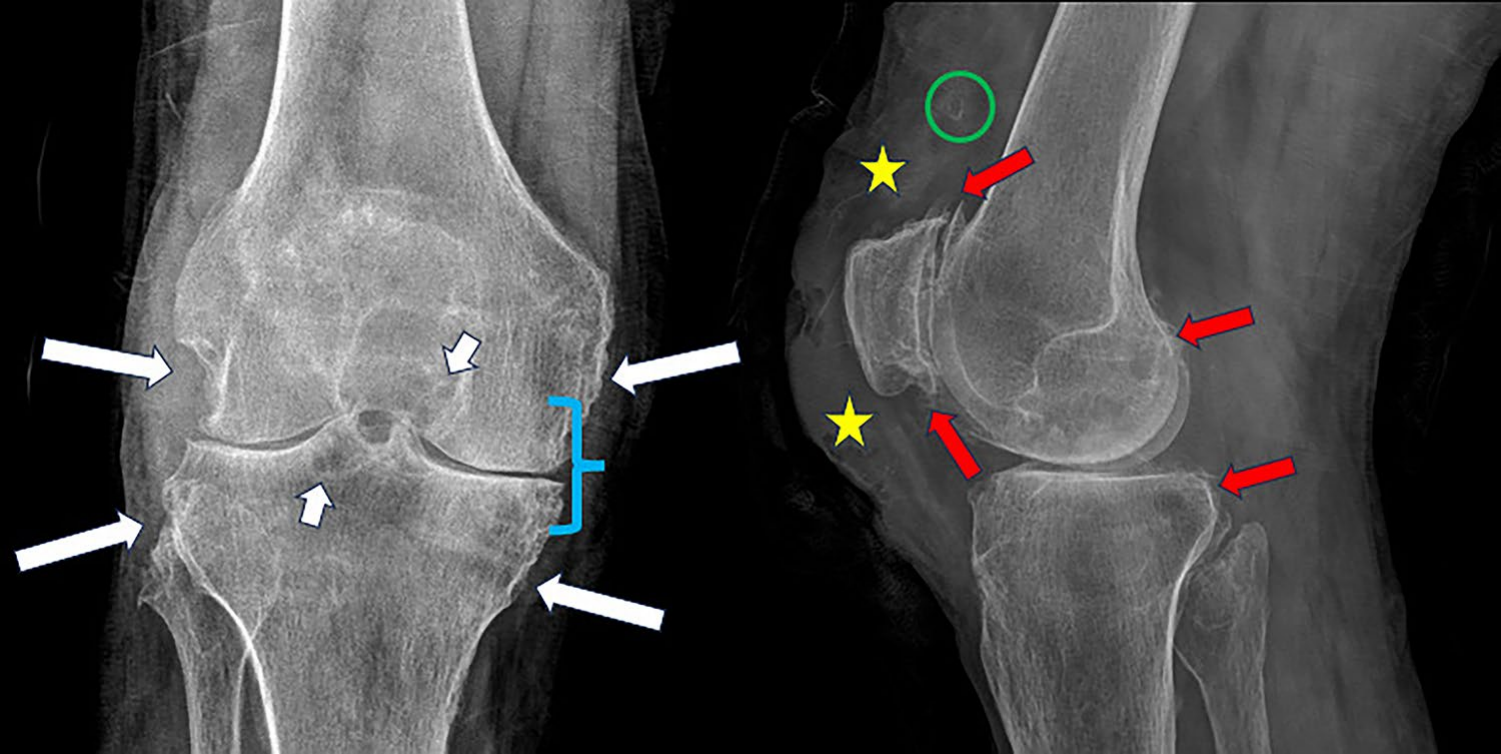
Radiograph of the right knee, anteroposterior (AP) and lateral (L) views, in a 52-year-old man with a 10-year history of chronic tophaceous gout presenting with purulent material oozing from a cutaneous nodule on the right knee. ABCDE’S radiographic approach: **A**: Adequate alignment. **B**: Enthesophytes of the extensor mechanism of the patella (red arrows), sclerosis and subchondral cysts (short white arrows) and decreased bone density. **C**: Symmetrically decreased joint space and increased amount of suprapatellar bursa fluid (blue bracket). **D**: Single joint. **E**: Long, parallel, sclerotic, non-marginal femorotibial and peroneal erosions (long white arrows). **S**: Thickening of the subcutaneous cellular tissue (yellow stars) and calcification projected on the suprapatellar bursa (green circle)

Plain radiography has low S and SP for detecting acute gouty crisis, which presents with joint effusion and unilamellar periosteal reaction due to adjacent inflammation [13, 14]. Chronic tophaceous gout typically presents with asymmetric polyarticular involvement of the lower limbs, often involving the Achilles tendon, olecranon bursa and extensor mechanism of the knee. Tophi appear as subcutaneous nodules of variable morphology, usually opaque and sometimes calcified [13, 14], with non-marginal or juxta-articular erosions with sclerotic contours, long and parallel to the long axis of the diaphysis [28] (Fig. 1).

**Fig. 2:**
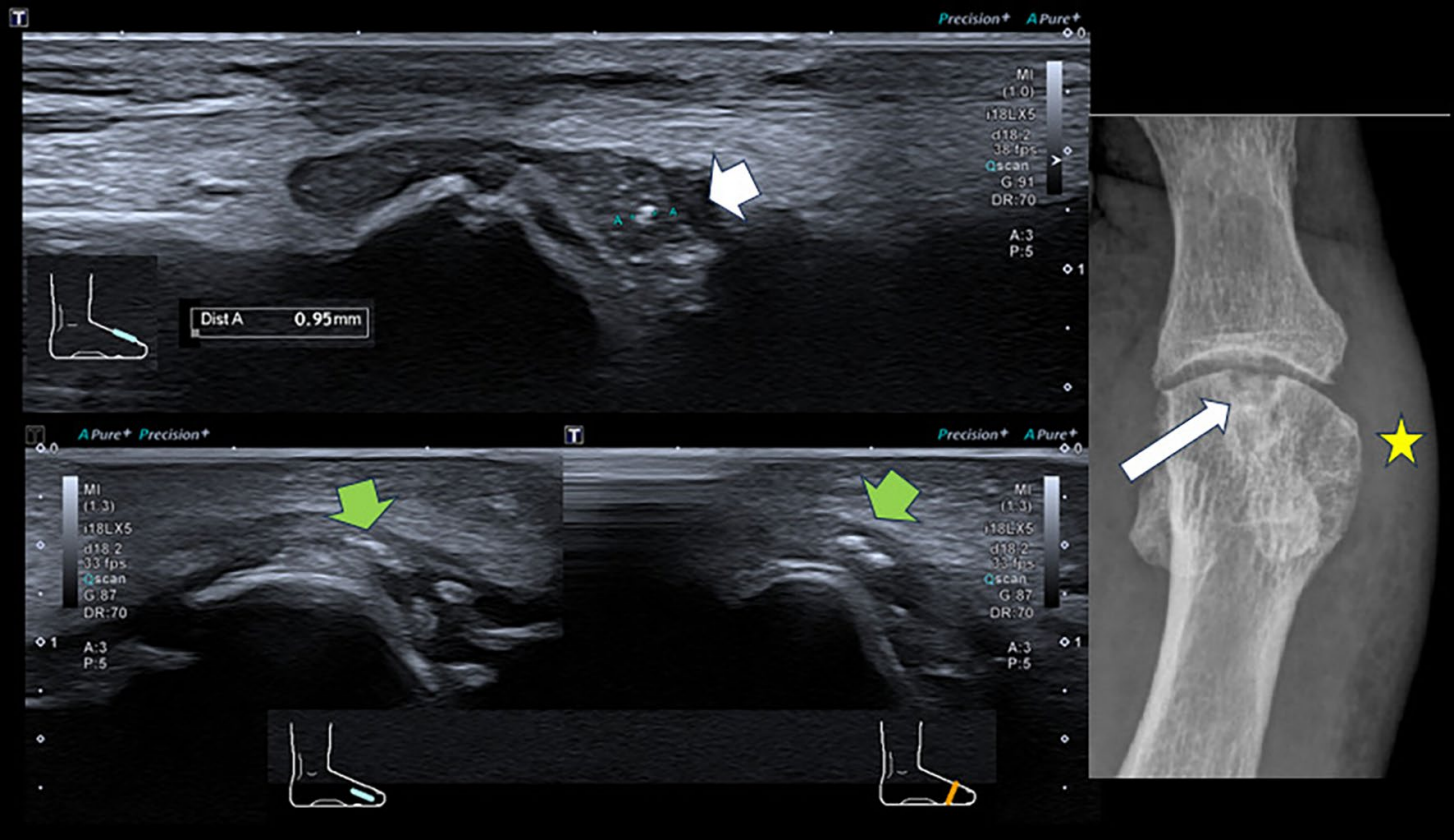
B-mode ultrasound images using an 18 MHz linear transducer and AP x-ray magnification of the first metatarsophalangeal joint of the first toe of the left foot in a 45-year-old man with a history of chronic gout presenting with an acute crisis. The long soft arrow shows a non-marginal erosion of the metatarsal head, which on ultrasound shows joint effusion, synovial thickening and internal mobile particulate content forming the ‘starry sky’ sign compatible with urate crystal deposits. The yellow star shows oedema of the superficial periarticular soft tissues

Ultrasound in acute gouty crisis shows the ‘double contour’ sign, a hyperechoic band of variable thickness on the articular cartilage, the ‘snowstorm’ sign with hyperechoic dots within an accumulation, and the ‘starry sky’ sign with articular effusion and internal hyperechoic foci less than 1 mm, together with findings of synovitis and bursitis [14, 20, 28] (Fig. 2). In chronic tophaceous gout, ultrasound shows tophi as well-demarcated, heterogeneous hypo-hyperechoic nodules with hyperechoic internal echoes, resembling ‘sugar lumps’ and possibly with posterior acoustic shadow [14, 20, 28]. Erosions are intra- or extra-articular with discontinuity of the cortex seen in two planes [14, 20, 28] (Fig. 2).

Dual-energy computed tomography is indicated for diagnosis and follow-up, with S of 78–100% and SP of 89–100% [24]. It differentiates calcium from monosodium urate crystals using colour mapping [16], and the protocol includes the symptomatic joint, usually extending to the hands and feet if gout is suspected [16]. Although less useful in the early stages, it is the gold standard in chronic disease and characterises bone erosions and tophi seen at a density of around 160 HU [16]. However, it does not detect low density crystals or crystals less than 2 mm in diameter.

**Fig. 3:**
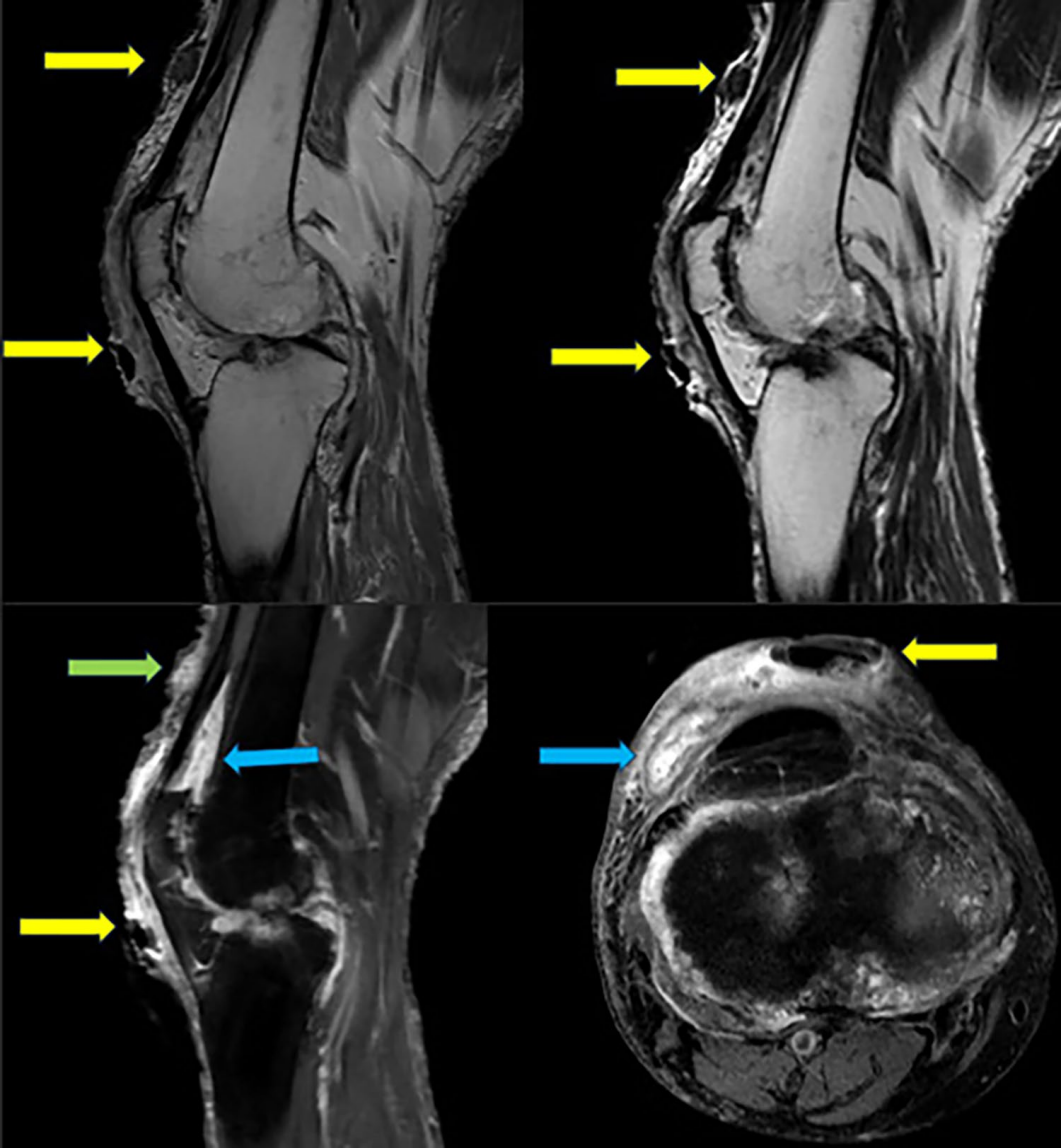
Magnetic resonance images of the right knee, sagittal proton density, T2, T1 with contrast and SPAIR axial slices, of the patient described in Figure 1, where the long yellow arrows show low signal nodules without contrast enhancement located in the prepatellar soft tissues, and in particular one with contrast enhancement in the prefemoral region (long green arrow). The blue arrows show thickening and enhancement of the synovial membrane of the suprapatellar bursa with internal particulate effusion. Findings compatible with subcutaneous gouty tophi and findings due to acute synovitis

Magnetic resonance imaging evaluates only the symptomatic joint, including anatomical, fluid-sensitive and dynamically contrasted sequences [16]. In a gouty crisis, MR identifies bone marrow oedema, soft tissue oedema, joint effusion with internal particulate material, and synovitis (Fig. 3). In chronic gouty tophi, tophi appear as amorphous or nodular areas of low signal on T1, with variable signal on T2 (particularly isointense or hyperintense) and variable enhancement with contrast [16] (Fig. 3).

### Calcium pyrophosphate deposition disease (CPPD)

#### Definition, epidemiology and pathophysiology

Inflammatory arthropathy caused by the deposition of dehydrated calcium pyrophosphate crystals in and around the joints. It has three stages: chondrocalcinosis, pseudogout and pyrophosphate arthropathy, defined by the identification of calcium deposits by imaging/histology in hyaline or fibrous cartilage, clinical arthritic attacks and structural bone and cartilage changes due to calcium, respectively.

There is no gender predilection and the prevalence increases with age [2]. The pathophysiological mechanism is unknown, but it is thought that traumatic cartilage damage, ageing [2] or a metabolic disorder leads to excess pyrophosphate crystals, articular cartilage loss, local inflammation and synovitis [14, 30, 31].

#### Clinical presentation and general diagnostic approach

Mainly mono or oligoarticular presentation. EULAR (European League Against Rheumatism) describes 4 types of CPPD [30]; asymptomatic, which is the most common (10–20%) [14], associated with osteoarthritic changes, acute arthritis and chronic arthritis [30]. Definitive diagnosis requires identification of crystals by polarising microscopy. However, any patient over 65 years of age with clinical signs suggestive of inflammatory arthritis in non-weight-bearing joints other than the knee is highly suggestive of the disease.

#### Imaging approach

**Fig. 4:**
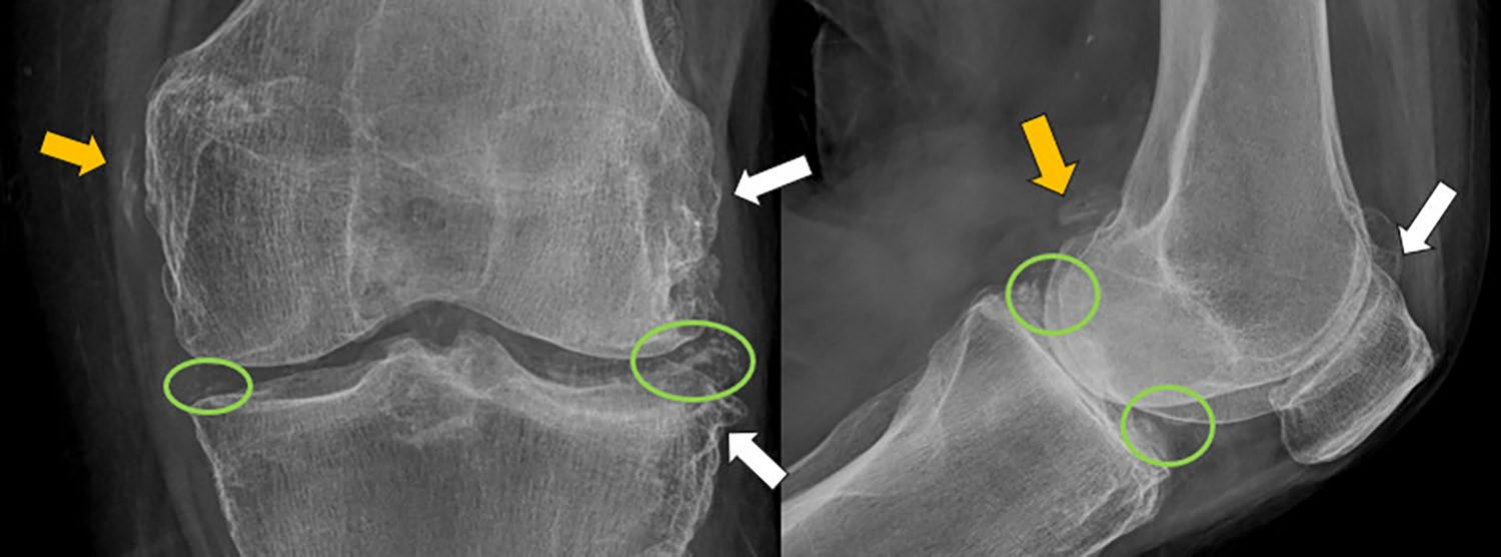
AP and L views of the right knee in a 60-year-old woman with left-sided gonalgia associated with increased joint volume and documented signs of chondrocalcinosus and pyrophosphate arthropathy. Radiographic ABCDE’S approach: **A**: Adequate alignment **B**: Marginal osteophytes of the outer contours of the femoral condyle and lateral tibial plateau (white arrows). **C**: Radio-opaque punctate foci of lesser density than the bony cortex, located 1-2 mm from the bony cortex, located in the topography of the medial and lateral meniscus (green circles). **D**: Decreased femorotibial joint space, predominantly left. **E**: No erosions are observed **S**: Radio opaque cottony foci compatible with calcification of the fascia lata tendon and gastrocnemius muscle (orange arrows)

The imaging approach for chondrocalcinosis and pseudogout has been updated to align with the ACR (Rheumatology) and EAAR consensus CPPD 2023. Radiography remains the first-line modality, identifying thin, continuous or discontinuous, linear or patchy calcifications in the medial cartilage, typically 1–2 mm from the cortex [13, 14, 17]. These calcifications are common in the menisci of the knee, the triangular fibrocartilage of the wrist, the labrum of the shoulder and hip, and the symphysis pubis, and often appear as cloudy opacities in the synovial membrane or para-articular soft tissues, including tendons, bursae and ligaments [13, 14, 17]. Radiographic features of pyrophosphate arthropathy include involvement of the wrist, metacarpophalangeal joint, talocalcaneal–navicular joint, or elbow, with severely reduced joint space with few marginal osteophytes, subchondral cystic degeneration (geodes) and sclerosis without erosions, and free intra-articular bony bodies in the synovial membrane [13, 14, 17] (Fig. 4).

**Fig. 5:**
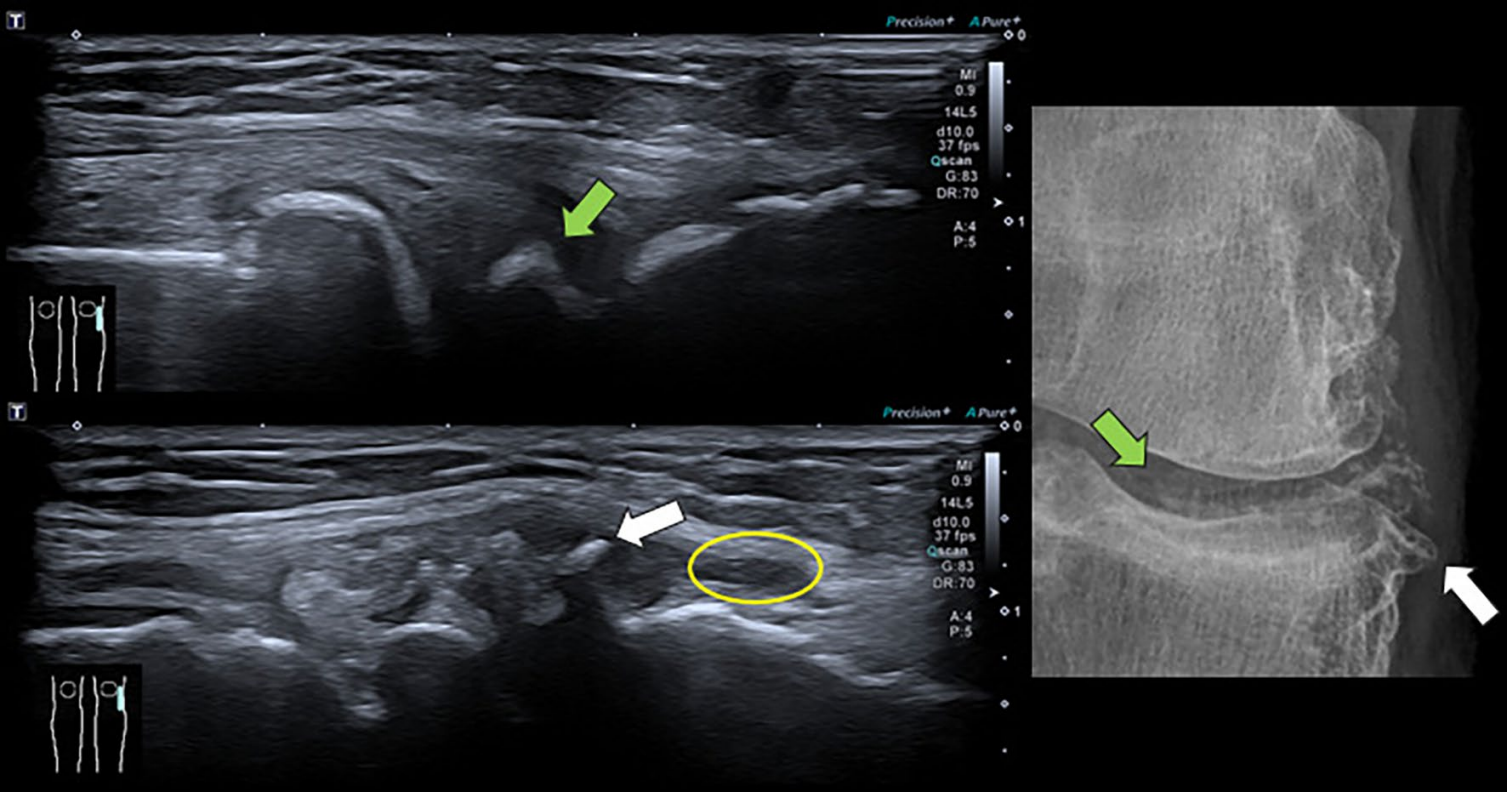
FiB-mode ultrasound images with 18 MHz linear transducer and AP radiographic magnification of the most superficial sector of the external compartment of the right knee of the previous patient, showing, with green arrows, a linear arc-shaped echogenic deposit with posterior acoustic shadow, located in the centre of the lateral meniscus of the knee, whose presentation in the radiographic magnification is compatible with the deposit of the pyrophosphate crystal, the most specific finding for the disease. The white arrows indicate the marginal osteophytes, particularly of the lateral tibial plateau, and the yellow circle indicates the synovial effusion and thickening. Findings compatible with ultrasound manifestations of chondrocalcinosis and pyrophosphate atropathy

Ultrasound shows irregular linear or punctate echogenic crystal deposits in the center of the cartilage, often without posterior acoustic shadowing, highly specific for pyrophosphate arthropathy and associated with loss of cartilage thickness and intra-articular bodies [17, 28, 34]. These findings extend to the synovium, capsule, ligaments and tendons with inflammatory changes, forming the ultrasound sign of pseudo double contour (Ps-DC), as the crystalline deposits are also seen superficial to the hyaline cartilage, but during dynamic manoeuvres they move in the opposite direction to the cartilage, a characteristic finding that can differentiate it from monosodium urate deposition disease. [17, 28, 34, 39] (Fig. 5).

**Fig. 6:**
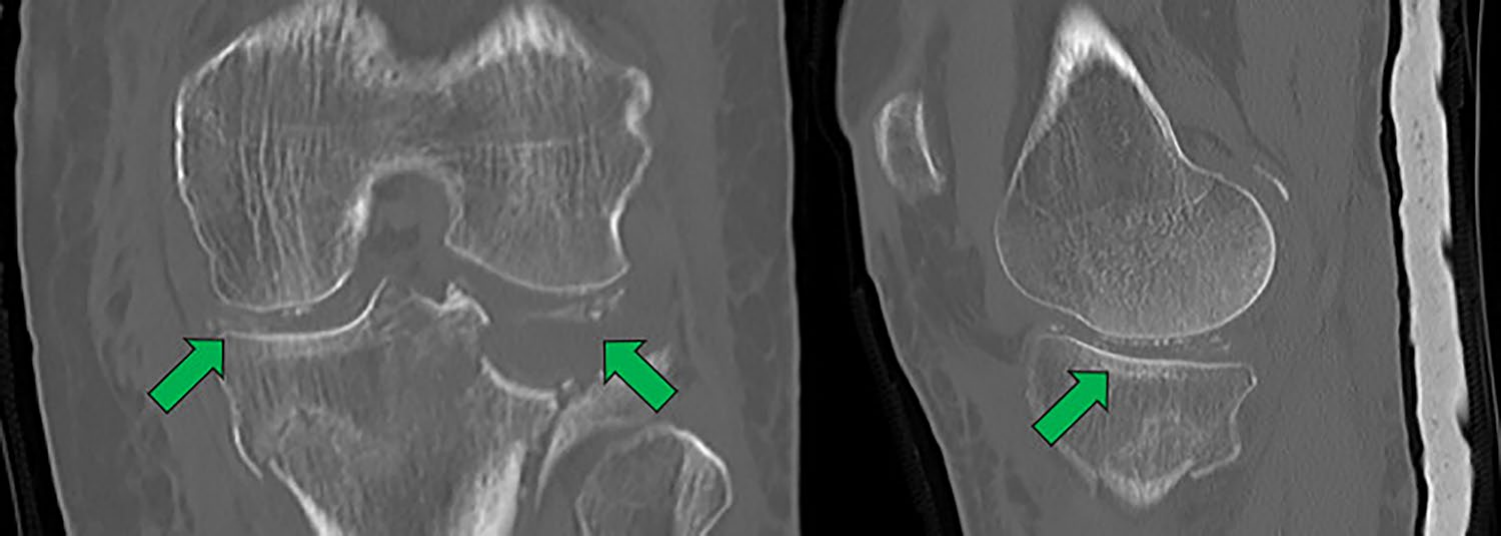
Enlargements of single tomography images of the right knee, coronal and sagittal reconstructions in bone window, in a 70-year-old patient who suffered a blunt trauma, where the green arrows show linear and some punctate calcifications with a density lower than the adjacent bone cortex, located in the medial and lateral menisci, as well as in the articular cartilage of the femoral condyle, as signs of incidental chondrocalcinosis. As an additional finding, the patient had a depressed fracture of the lateral tibial plateau requiring intervention

Conventional tomography shows linear or punctate calcifications of lower density than the bone cortex in hyaline or fibrous cartilage, with similar features in the synovial membrane, capsule, tendons, ligaments or bursae, manifesting as cloudy or hazy opacities in acute crises [16, 17] (Fig. 6).

Magnetic resonance imaging detects cartilage calcifications by their low signal in all sequences, although it is more difficult to identify deposits in ligaments [16, 17].

### Basic calcium deposition disease—hydroxyapatite arthropathy

#### Definition, epidemiology and pathophysiology:

Deposition of basic calcium, hydroxyapatite, octacalcium, tricalcium and whitlockite, in periarticular tissues, mainly in tendons and bursae [14], or in intra-articular locations, most commonly in rotator cuff tendons, gluteal muscles and rectus femoris muscles [2, 4, 13]. It affects adults aged 30–60 years, with a predilection for women, with an approximate prevalence of 3–8% in asymptomatic individuals and up to 42% in subacromial pain [4].

The pathophysiology remains unknown. It is thought to occur due to degenerative causes, ischaemia, trauma, chondrocyte mediated formation or endochondral ossification [25].

Uhthoff and Sarkar described three phases in the hydroxyapatite disease process; the pre-calcifying state, the calcifying state with its subdivisions of formative, quiescent and resorptive, and the post-calcifying state.

During the calcifying-resorptive phase, minor trauma or overuse of the joint can cause existing calcium crystals to become detached and shed into the surrounding tissue, leading to local inflammation, cartilage destruction, synovitis and chondrocyte apoptosis. This process is known as periarthritis or acute calcific arthritis and Milwaukee shoulder [4, 6, 25].

#### Clinical presentation and general diagnostic approach

Basic calcium deposition disease may present as asymptomatic calcifying tendinitis or Milwaukee shoulder [2]. The disease is predominantly monoarticular [14], most commonly affecting the shoulder, followed by the hip, wrist, hand, spine and neck [2, 4, 6].

Definitive diagnosis is made by polarising microscopy. However, the diagnostic approach in patients under clinical suspicion should include a clinical history, imaging studies and synovial fluid analysis [2, 4, 6]. In contrast, the diagnosis of Milwaukee shoulder syndrome must meet all of the following requirements [32]: Osteoarthritic changes, subchondral and cartilage destruction, non-inflammatory joint effusion containing calcium pyrophosphate crystals and synovial hyperplasia, multiple osteochondral free bodies, and ruptures of the rotator cuff.

#### Imaging approach

**Fig. 7:**
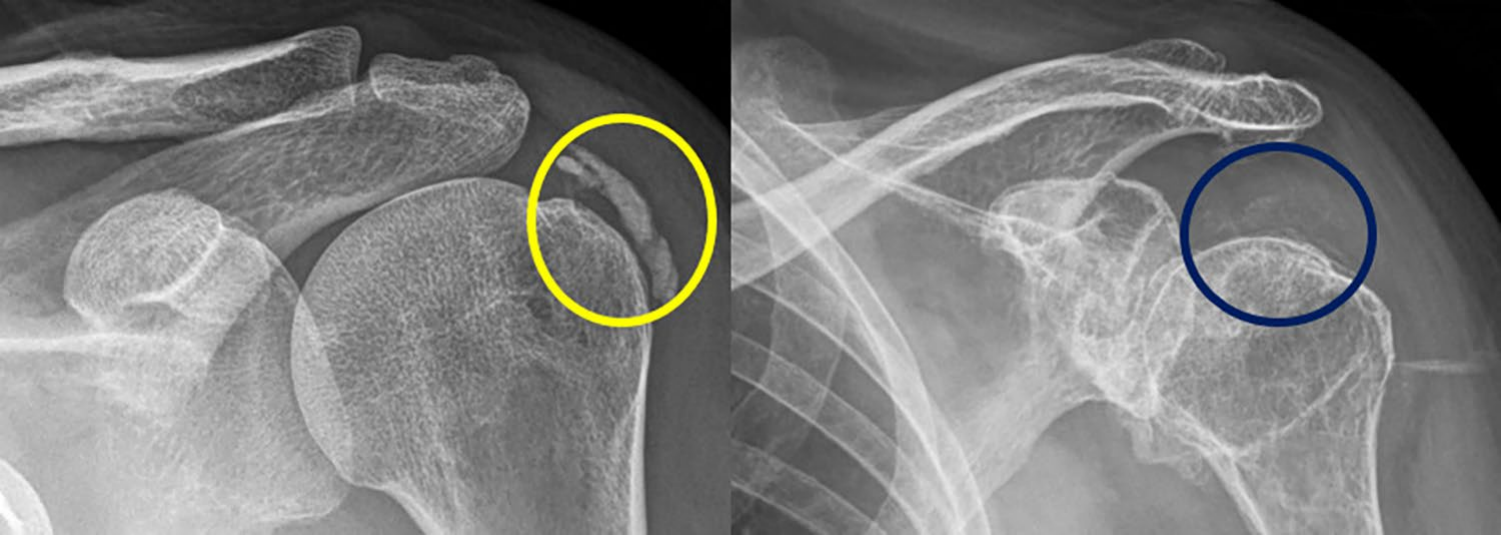
Magnification of plain radiographs of the left shoulder, AP projections, in two different patients with acute shoulder pain. The left image, surrounded by a yellow circle, shows an area of dense calcification with well defined contours in the topography of the supraspinatus tendon. This finding is compatible with type I calcific tendinosis in the early stages. In the image on the right, surrounded by a blue circle, there is another area of less dense calcifications with poorly defined contours, also in the topography of the tendon of the supraspinatus muscle. This finding is compatible with calcific tendinitis in the reabosorptive phase

**Fig. 8:**
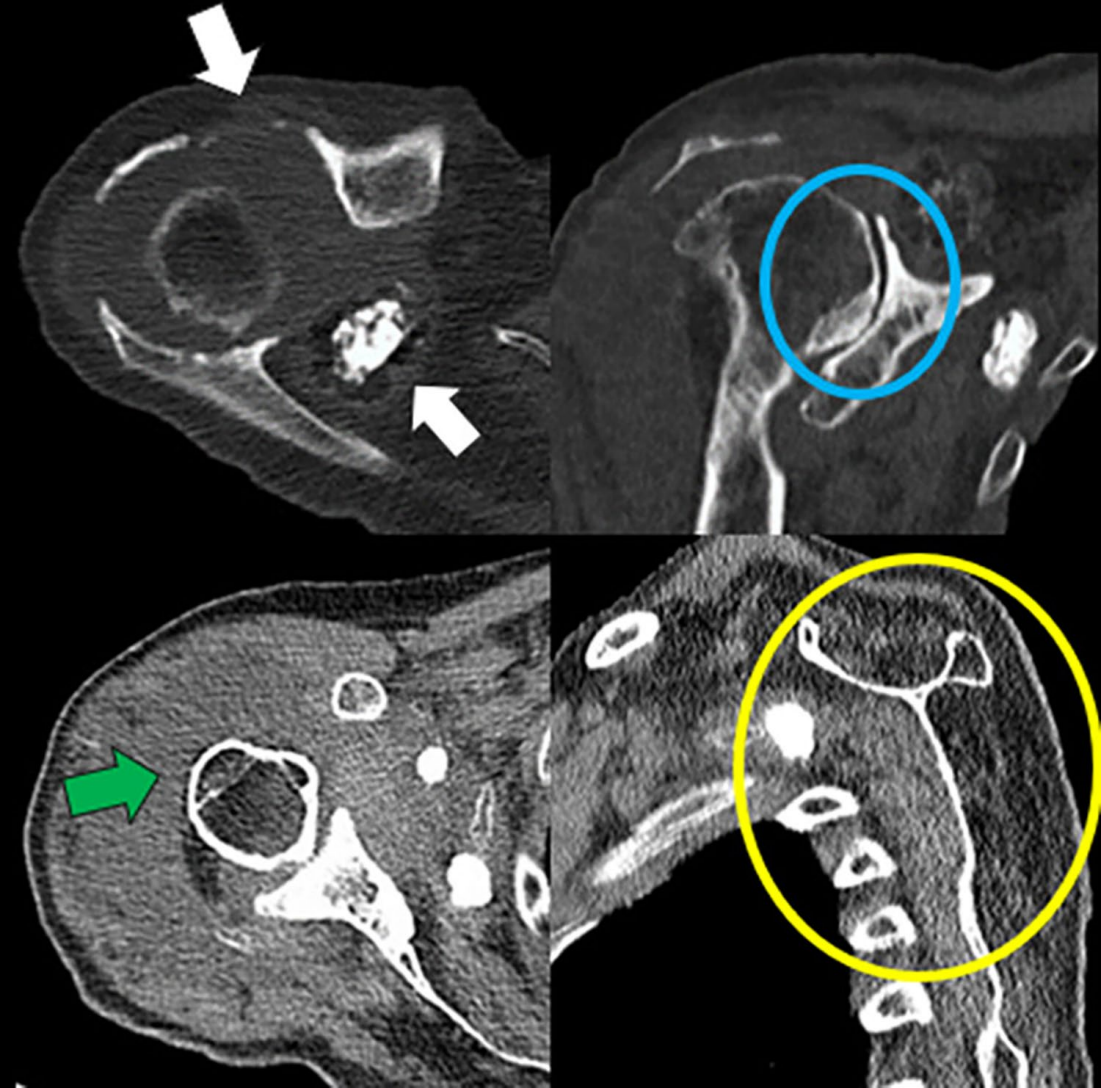
Plain tomography images of the right shoulder, axial images and multiplanar reconstructions, in the bone and soft tissue window, of an older adult patient with mild chronic pain of the right shoulder.The white arrows indicate bone destruction of the acromioclavicular joint and an intra-articular free bone body. The blue circle shows the ascending humeral head with decreased glenohumeral joint space with subchondral sclerosis. The green arrow shows increased volume and effusion of the subacromio-subdeltoid bursa, and the yellow circle shows fatty infiltration and atrophy of the supraspinatus and infraspinatus muscles as indirect signs of rotator cuff tear. Overall, the findings are consistent with advanced Milwaukee’s shoulder

Radiographically, calcific tendinitis presents as heterogeneous, ill-defined or cloudy, opaque radiopaque images in its acute form, and homogeneous, discrete and well-defined in its chronic form, typically without bone erosions [4, 6, 25, 32]. These calcifications are usually located in the critical area of tendons, tendon sheaths or bursae [13], with the supraspinatus tendon being the most common site in the shoulder (80%) [13]. In the hip, calcifications are found at the insertion of the gluteus maximus and medius tendons over the greater trochanter and its adjacent bursa [13]. Gartner and Heyer classified calcifications according to size, morphology and density [14] (Fig. 7), specifically describing the calcification phase as type I (formative, asymptomatic: dense calcifications with defined borders), type II (quiescent: may be dense/radiolucent with defined/undefined borders) and type III (resorptive, symptomatic: radiolucent calcifications with undefined borders). In the resorptive stage, calcifications may migrate into soft tissues such as tendons, bursae, joints or bones [13, 14], requiring US or MRI for identification [14]. Milwaukee Shoulder presents with intra-articular calcifications migrating from calcific tendinitis in the resorptive phase [25], leading to joint destruction. Early findings include reduced joint space, ascending subluxation of the humeral head, sclerosis of the articular surfaces, subchondral cysts and erosions of the greater tuberosity [4, 6, 25, 32]. More advanced findings include severe destruction of the glenohumeral joint, bony and cartilaginous sclerosis, subchondral cysts, intra-articular free bony bodies, bursa and tendon calcifications, soft tissue oedema, severe damage to surrounding bone and formation of new joints or pseudojoints [13] (Fig. 8).

Ultrasound in acute calcific tendonitis (resorptive phase) may show nodular (single echogenic focus without posterior opacity), fragmented (more than 2 echogenic foci without posterior opacity) or cystic (echogenic wall with anechoic or hypoechoic particulate content with flattening) presentations, with increased vascularity on Doppler differentiating it from degenerative calcifications seen in tendon rupture [4, 25]. In chronic calcific tendinitis (formative/relapsing phase), echogenic arches are well defined and cause posterior acoustic shadowing [4, 25]. Milwaukee shoulder ultrasound shows rotator cuff tears, joint effusion, synovitis, calcium deposits and osteolysis [32].

**Fig. 9:**
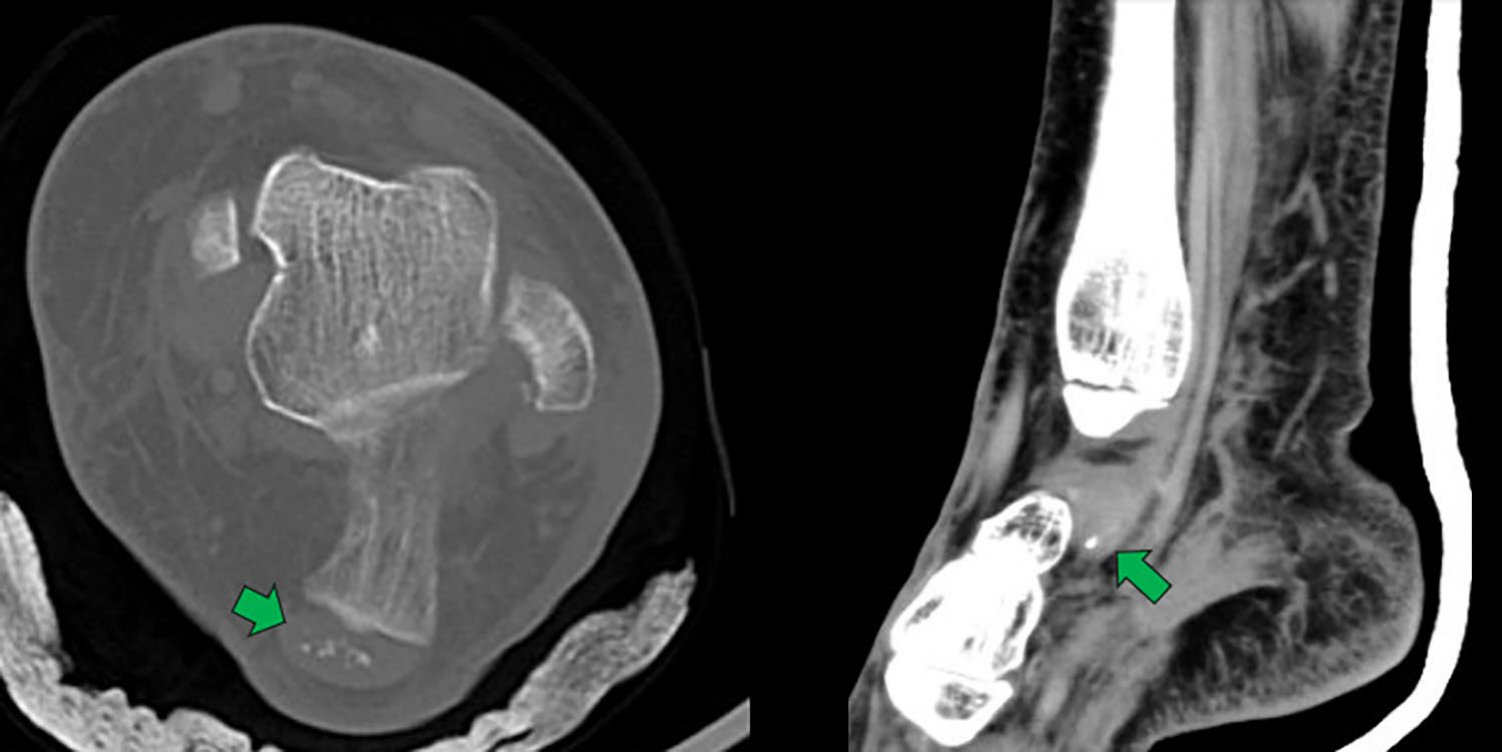
Single tomography images of the left ankle, axial acquisition in the bone window and coronal reconstruction in the soft-tissue window, of a 56-year-old female patient who suffered an inversion trauma, detailed with green arrows pointing to calcium deposits in the centre of the Achilles tendon and along the tendon of the posterior tibial muscle, forming the comenta tail sign

CT scan shows similar signs to conventional radiographs, but can differentiate basic calcium calcifications from mature bone by their lower density; basic calcium (100–400 HU) and cortical bone (700–1500 HU). The comet tail sign shows diffuse calcium deposits along the length of the affected tendon in both its formation and resorption phases [4, 16, 25] (Fig. 9).

Magnetic resonance imaging [16] is useful in identifying migration of calcium deposits into surrounding tissues and secondary inflammation, as well as other soft tissue changes and articular cartilage involvement [16, 25]. Deposits are seen as areas of low intensity on T1 and T2 sequences [25].

### Neuropathic arthropathy of the foot or charcot’s arthropathy

#### Definition, epidemiology and pathophysiology

Neuropathic arthropathies are defined as a group of diseases involving joint destruction and atrophy with concomitant bone formation due to multiple factors, including peripheral neuropathy, trauma and alterations in bone metabolism, often associated with patients with diabetes mellitus and peripheral neuropathy [33]. The most commonly affected parts of the body are the foot and ankle, but any joint such as the hip, shoulder, hands and spine can be affected [34]. Neuropathic arthropathy of the foot or Charcot foot has a prevalence of 0.4–13% in the population with diabetes mellitus and 9% in patients with foot ulcers [35].

The pathophysiology of the disease is unknown, but two possible pathophysiological mechanisms have been proposed. The neurotraumatic theory suggests that acute-subacute repetitive trauma in the absence of a protective pain stimulus maintains a proinflammatory state in soft tissues and bone, activating osteoclasts and causing disproportionate bone resorption [7]. The neurovascular theory posits that autonomic hyperactivity causes vasodilatation and hyperemia, leading to increased venous pressure, leakage of fluid through capillaries into interstitial tissues, increased compartment pressure, and deep tissue ischaemia [7]. Hyperemia promotes bone resorption, and ischaemia weakens tendons and ligaments, leading to instability [7].In both theories, incorrect weight bearing without protective proprioceptive mechanisms to redistribute weight creates microtrauma, triggering pro-inflammatory processes, pathological bone remodelling and joint instability [7].

To understand the pathophysiology, stages in the disease continuum have been described using the modified Eichenholtz classification for the foot and ankle, as shown in Table 2 [7]:

**Table 2:** Modified Eichenholtz classification for the foot and ankle

	Stage 0	Stage 1 Fragmentation or resolution phase	Stage 2 Coalescence or early healing phase	Stage 3 Reconstruction phase
Clinical	There is oedema and erythema with loss of paresthesia. Clinical instability is present	Oedema and erythema persist. Ligament hyperlaxity increases	Decrease in oedema and erythema	Absence of swelling. Foot more stable but deformed
Radiographic	usually normal	Osteopenia, periarticular fragmentation, fractures and/or subluxations	Resorption of bone debris, early fusion of fragments and sclerosis	Arthrosis, osteophytes, subchondral sclerosis and deformity

#### Clinical findings and general diagnostic approach

Patients present with two clinical pictures. The first is acute neuropathic arthropathy, which presents with erythema, warmth, flushing and oedema of the foot or ankle with preserved pulses (any joint), associated with dull, ill-defined or painless pain. There is no constitutional syndrome. Varying degrees of instability and a rocker deformity with valgus angulation of the foot may be observed as the most common deformity [36]. Another possibility is chronic neuropathic arthropathy, where there is a long-standing joint deformity that may be associated with ulceration or osteomyelitis [36]. Diagnosis is made in a patient with neuropathy or long-standing deformity confirmed by imaging [33, 35]. The diagnostic approach is based on a suggestive clinical presentation in a patient with a history of diabetes mellitus and peripheral neuropathy, supported by imaging and laboratory studies [33, 35].

#### Imaging approach

**Fig. 10:**
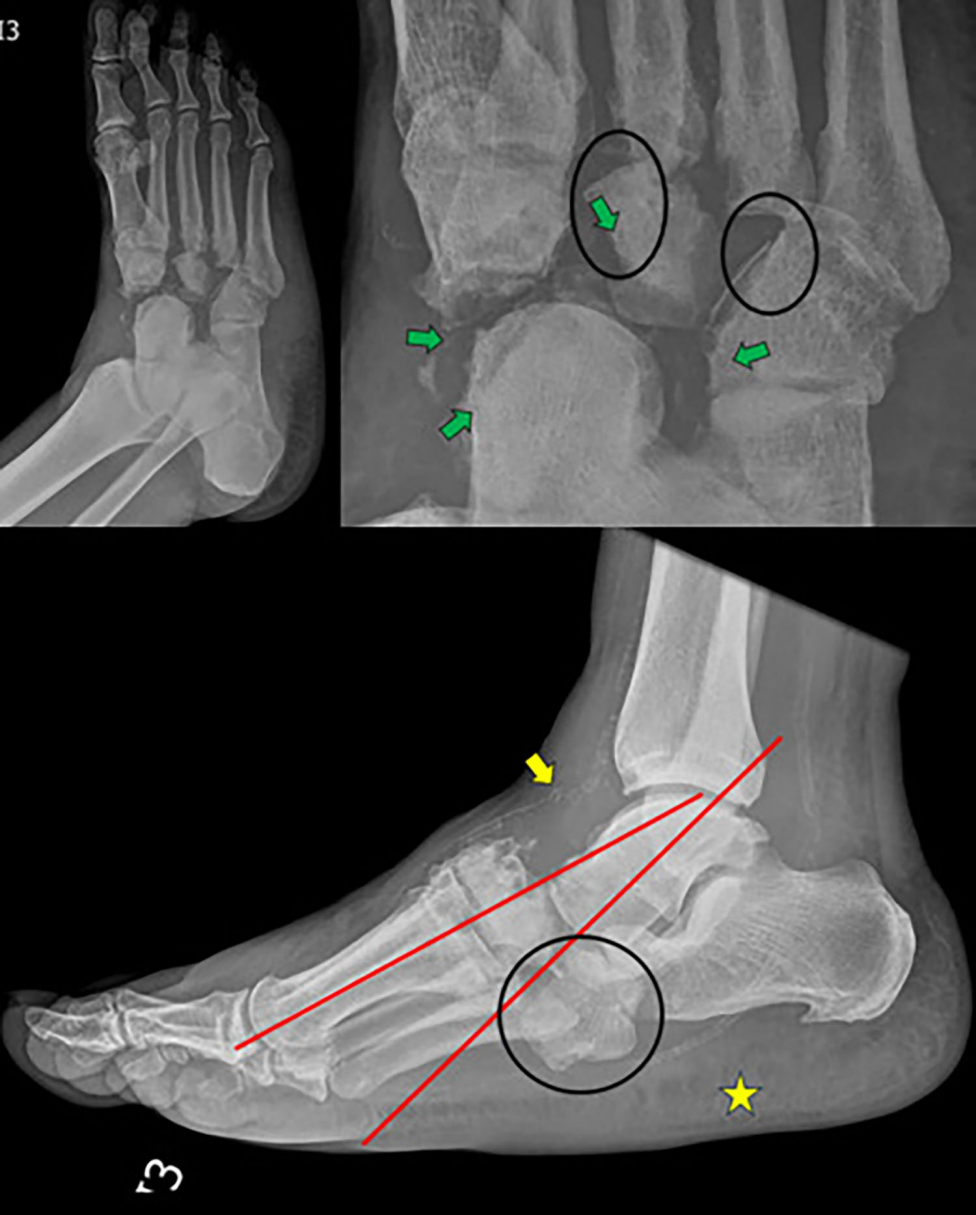
Conventional radiographs of the left foot in oblique projection, magnification of the oblique projection with emphasis on the midfoot, and lateral projection of a 46-year-old man with a history of type 2 diabetes mellitus who presented with 15 days of right foot pain associated with a plantar lesion. The patient had findings compatible with neuropathic atropathy of the foot or Charcot’s atropathy in the fragmentation phase or acute neuropathic arthropathy. The following is the ABCDE systematic approach to plain radiography: **A**: Subluxation of the talonavicular joint and 3rd wedge cuboid metatarsal (straight red lines). **B**: Focal osteopenia of the midfoot, subchondral cysts and large osteophytes (surrounded by black circles). **C**: Intra-articular bony bodies (indicated by green arrows). **D**: Midfoot involvement. **E**: Erosions. **S**: Soft tissue oedema (indicated by the yellow star). Calcification of the pedicle artery (indicated by the yellow arrow) is also seen

**Fig. 11:**
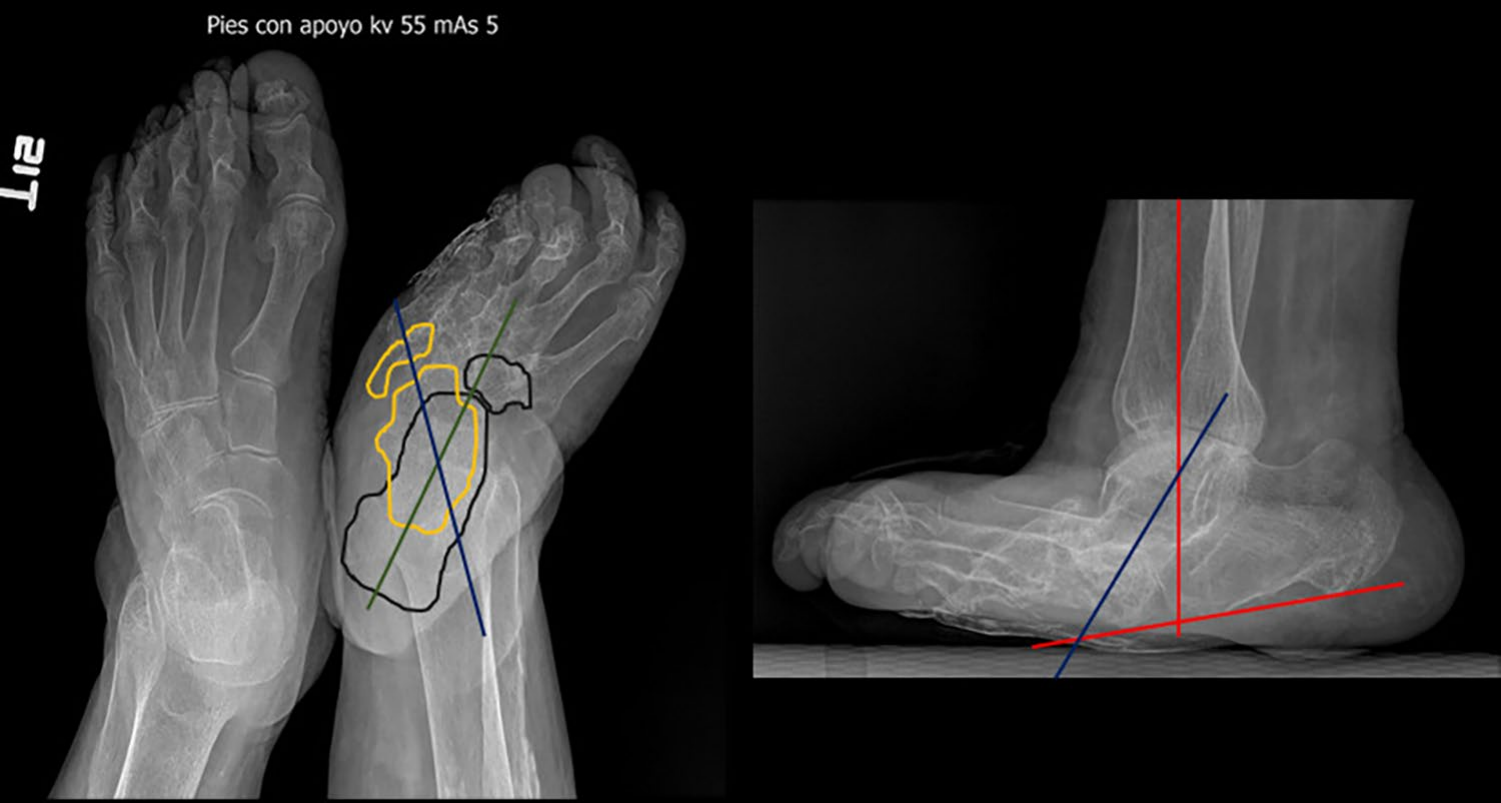
Comparative plain foot radiographs, anteroposterior projection and right foot radiograph with lateral projection support, of an 86-year-old patient with complex regional pain in the left foot associated with chronic osteomyelitis of the talus, tibial malleolus and distal metaphysis of the right tibia since 2016 with amputation of the 1-2 artery in 2017. Findings of the right foot: Changes due to chronic hypertrophic neuropathic arthropathy in the coalescence or remodelling phase. Absence of the phalanges and metatarsals of the first toe. **A**: Calcaneal deformity of the hindfoot in front of a tibiocalcaneal angle of less than 60° (see-saw deformity). Talonavicular subluxation (bones marked in yellow and joint axis marked in blue). Cuboidal calcaneal subluxation (bones outlined in black and axis outlined in green). Varus deformity of the 3rd and 4th metatarsophalangeal joints. **B**: Diffuse decreased bone density, sclerosis and subchondral cysts and marginal osteophytes of the forefoot, midfoot (predominantly) and hindfoot joints. **C**: Ankylosis of the wedge-metatarsal and cuboid-calcaneal joints. **D**: Mainly distributed over the midfoot and hindfoot. **E**: No erosions. **S**: Oedema of the superficial soft tissues.

Radiography is the initial and follow-up modality for Charcot foot, with low sensitivity and specificity for the diagnosis of osteomyelitis [18, 34]. Supported projections improve the detection of small fractures, fragments, subluxations, joint deformity or collapse. The earliest finding is demineralisation often with flattening of the metatarsal head as the first sign of diabetic neuroarthropathy [19]. In acute arthropathy, radiographs may initially be normal, with midfoot involvement being the most common and initial signs including flattening of the metatarsal heads [34]. Adequate alignment is maintained until loss of joint relationship. In the acute phase there is decreased bone density, fractures, subchondral cysts, intra-articular free bone bodies and increased joint fluid associated with soft tissue oedema [34]. Chronic arthropathy at follow-up shows disappearance, fusion and ovalisation of longer bone fragments, bone sclerosis, consolidation of fractures and a hypertrophic pattern with cartilage destruction, bone debris, joint dislocation and destruction, joint distension and normal/increased bone density [19]. Osteophytes differ from those of osteoarthritis by early formation of ill-defined or rounded contours and large size, together with joint disorganisation and joint blood leakage. Severe advanced osteoarthritis is almost indistinguishable [37] (Figs. 10, 11).

Ultrasound is indicated as an adjunctive study to detail soft tissue involvement, but does not complement destructive and hypertrophic bone changes [18, 19].

Computed tomography is generally not indicated for diagnosis due to its inability to detect microfractures and bone oedema [7, 19], but may be useful in the preoperative evaluation to detail bone erosions and sclerosis [18, 19].

Magnetic resonance imaging is initially indicated to rule out differential diagnoses such as osteomyelitis or infection superimposed on neuropathic arthropathy, with an S of 90% and SP of 79% [18]. When in doubt, fluorodeoxyglucose positron emission tomography labelled leukocytes is recommended, as it is more sensitive than contrast-enhanced MR [38], with combined results from both modalities approaching 100% sensitivity. MR shows bone oedema, erosions, subchondral cysts, intra-articular free bodies, joint effusion and inflammatory soft tissue changes. It is important to differentiate the changes expected in neuropathic arthropathy of the foot from osteomyelitis; diabetic foot oedema occurs in the periarticular areas of the midfoot, tarsometatarsal or metatarsophalangeal areas, associated with subchondral cysts and intra-articular bodies [18]. Synovial or bursal diverticula mimicking abscesses may also develop [18]. In scenarios where osteomyelitis is superimposed on neuropathic arthropathy of the foot, characteristic signs include the phantom sign, bone marrow changes such as T2 enhancement and enhancement beyond the joint surfaces, progressive bone resorption, increased periarticular enhancement, midfoot involvement, synovitis, erosions of intra-articular bony bodies and subcutaneous inflammatory changes forming abscesses, sinus tracts and ulcers [18].
